# Anxiety disorders among children and adolescents during COVID-19 lockdowns and school closures: a cross-sectional study in Kuwait

**DOI:** 10.3389/fpsyt.2024.1322745

**Published:** 2024-02-12

**Authors:** Bibi Alamiri, Moh A. Alkhamis, Ahmed Naguy, Hend F. Alenezi, Muna Al Shekaili

**Affiliations:** ^1^ Almanara, Kuwait Center for Mental Health, Ministry of Health, Kuwait City, Kuwait; ^2^ Department of Epidemiology and Biostatistics, Faculty of Public Health, Health Sciences Centre, Kuwait University, Kuwait City, Kuwait; ^3^ General Adult Psychiatry Department, Kuwait Center for Mental Health, Kuwait City, Kuwait; ^4^ Ministry of Health, Almassarh Hospital, Muscat, Oman

**Keywords:** anxiety, mental health, pandemic, children, COVID-19, lockdowns, school closures

## Abstract

**Introduction:**

Investigating the epidemiology of mental health disorders resulting from COVID-19 intervention measures, primary school closures, and social isolation in children and adolescents needs to be prioritized over adults at the post-pandemic stage. Most preliminary psychosocial studies conducted during the pandemic have demonstrated that younger age groups are the most vulnerable to such implications. Thus, this study aims to estimate the probable prevalence of specific anxiety disorders in children and quantify their relationships with relevant demographic risk factors.

**Methods:**

We used a cross-sectional study comprising 430 children aged between 8- and 18 years old living in Kuwait during the period of school closures as well as full and partial lockdowns. The survey included questions about participants’ characteristics, children's anxiety using the Screen for Child Anxiety Related Emotional Disorders Questionnaire (SCARED) scale, and children's emotions and behaviours using the Strengths and Difficulties Questionnaire (SDQ). Univariate and multivariate logistic regression analyses were used to summarize the demographic and characteristics of the participants and their association with general, social, and generalized anxieties, as well as behavioural and emotional difficulties.

**Results:**

We inferred that 24.83% of our participants had at least one anxiety disorder, while 20.19% were classified as abnormal on the SDQ scale. Our multivariate analysis revealed that lockdown duration and sex of the child were consistently significant predictors (p-values < 0.05) of the broad spectrum of selected mental disorders. Additionally, we inferred notable increases in the likelihood of mental disorders associated with the increased duration of lockdowns.

**Conclusions:**

Our findings revealed preliminary insights into the vulnerability of young populations to the indirect negative impacts of strict public health measures during pandemic emergencies. Thus, authorities should consider such implications when planning and implementing similar interventions in future pandemics.

## 1 Introduction

Severe acute respiratory syndrome coronavirus 2 (SARS-CoV-2) has posed an unparalleled threat to public health and the economy on a global scale. In addition to the physical health implications of COVID-19, past studies showed that non-pharmaceutical interventions such as lockdowns, social distancing, school closures, and self-isolation were also detrimental to the mental health of the general population, particularly adolescents and children (1, 2). This is not surprising since the COVID-19 pandemic has already been characterized as a mass trauma event by the international communities for trauma research. This is reflected by the fact that it suddenly forced populations to change the fundamental features of their conventional anthropological nature (3, 4). Additionally, the prolonged enforcement of public health interventions (*i.e.*, for approximately two years) demanded remarkable changes in behavioral and cognitive aspects of daily life, resulting in a noteworthy increase in mental health illnesses surpassing the direct physical effects of COVID-19 infections (4, 5).

Stressors leading to mental disorders such as anxiety and depression resulting from the prolonged implementation of intervention measures may include a fear of being infected, frustration, boredom, inadequate supplies, lack of information and uncertainty, financial loss, and stigma (6, 7). However, studies have shown that while anxiety and depression rates were highest at the beginning of the implementation of lockdown measures, they both showed a stable decline over time, particularly among adolescents (5, 8). That said, identifying high-risk groups is critical in understanding the epidemiology of anxiety and depression in susceptible populations. For example, it was found that females reported notably higher rates of psychological distress during the early phase of the pandemic than males (3, 8). Additionally, depression and anxiety rates were higher among young adults aged less than 35 compared to older age groups in the United Kingdom (9, 10). Other risk factors identified during the pandemic included preexisting mental health conditions, socioeconomic status, employment status and self-reported loneliness (8–10).

Despite the remarkably lower hospitalization and death rates from COVID-19 among children (11, 12) due to the developing physiology of their immune system (13), they were the most vulnerable to the devastating psychological implications of the pandemic and intervention measures (14). Children start to be preceptive of the changes occurring in their environment by age two (15). Furthermore, children who stay at home away from their distant family members, friends, and school can experience fear, anxiety, distress, and social isolation due to the fear of infection and the uncertainties surrounding the pandemic status (14, 16). Additionally, the parents’ stress during the quarantine and social isolation at home can amplify the adverse psychological effects on children and lead to an abusive environment (17). This can lead to short or long-term effects that are detrimental to their brains’ growth and development (18). Common changes observed in children’s behavior include excessive crying, increased sadness, difficulties with concentration, and changes in activities they enjoyed in the past (19). Thus, pandemics can negatively influence children’s psychological health (20), leading to more significant percentages of anxiety and depression (21). In fact, a remarkable 81% increase in children and young people’s referrals to mental health services was observed in 2021 compared to 2019 (22). In addition, a global survey by the United Nations International Children’s Emergency Fund (UNICEF) revealed that 1 in 5 young people reported depression symptoms in 2021 (23). This high prevalence of anxiety and depression among children and young people has led to an estimated net loss to the global economy of approximately $390 billion a year since the start of the Pandemic (23).

While the role of school closures in reducing the community transmission of COVID-19 remains inconclusive (24), many studies suggested that such an intervention measure was a fundamental cause of mental illness among children and adolescents (25–29). In addition to the direct effect of interruptions in the education cycle, international educational organizations have highlighted their concerns about the long-term harmful effects of COVID-19 school closures on generations of students worldwide regarding mental health and economic implications (30, 31). This is not surprising since school environments are the cradles of social interaction between and among children and adolescents. Social exposure, particularly in school environments, is essential for the healthy development of children and youths’ mental and cognitive functions (29, 32). For example, it is known that anxiety-provoking and fear situations are critical components of the anxiety treatment (32), which were substantially limited to children and youths during the implementation of social restriction measures. Further, such measures also limited access to school services such as meals, physical activities, social consultation, and surveillance of children for neglect or abuse (28).

Despite the notable global drops in deaths and hospitalizations, the number of epidemiological studies investigating the short-term physical impacts of COVID-19 infection remains considerably more significant than its psychological impacts. Thus, local and regional observational studies on the short- and long-term adverse effects of COVID-19 intervention measures on the mental health of children and youths need to be prioritized over adults at the post-pandemic stage. This is because children’s symptoms of mental illness are often not observed or notably expressed like adults, and they have fewer experiences and resources to cope with stressful environments like the pandemic situation. In addition, it was shown that public health measures like quarantine and social isolation were significant predictors of future mental illness in children and adolescents (33). The few regional studies conducted in the Middle Eastern countries revealed a notable prevalence of anxiety and depression among children and young adolescents (34–37). These prevalences were either slightly higher or similar to what has been estimated elsewhere (38, 39) within few decades before the pandemic in the region. Here, we used a cross-sectional study to investigate the prevalence of anxiety disorders during the peak implementation of the pandemic intervention measures among children in the State of Kuwait. More explicitly, we estimated the prevalence of specific anxiety disorders in children and quantified their relationships with relevant demographic risk factors and the implemented public health measures. Here, we present the first epidemiological picture of different anxiety disorders in Kuwait. Thus, the findings of our study are critical for guiding the development and implementation of targeted post-pandemic psychological disorders intervention programs for high-risk groups.

## 2 Methods

### 2.1 Participants

We used an observational cross-sectional study comprising children aged between 8- and 18-years old living in the state of Kuwait between April 2020 and July 2020, covering the period of school closures and full and partial lockdowns screening for anxiety disorders. We selected our age group based on the requirements of the data collection instruments described below. The COVID-19 lockdowns in Kuwait started as partial lockdowns (or curfews) for 12 hours daily on March 22^nd^ 2020 (*i.e.*, between 5 pm and 4 am). Then, on May 10^th^, 2020, the partial curfew was transitioned to a full curfew until May 31^st^ 2020. The full curfew was followed again by partial curfew till April 2021. During the pandemic, schools in Kuwait were closed for in-person learning from the 1^st^ to 12^th^ grades between February 2020 and October 2021 (*i.e.*, approximately 18 months). Online schooling was implemented in August 2020, and it was a mixture of full and customized schedules depending on the type of school (e.g., private or public/primary or secondary).

We could not use, for example, a cluster sampling strategy through random selection of the schools due to COVID-19 closures and the limited availability of their electronic resources and contact information that may have facilitated the distribution of the questionnaires. We collected the data through an anonymous structured survey disseminated nationwide through local social media platforms (*i.e.*, WhatsApp and Instagram) using convenience and snowball sampling involving no contact with responders. Our final inclusion criteria included children between 8- and 18- years old, with a literate guardian(*i.e.*, the person who is legally responsible for the child’s care and filled out the questionnaire), living in Kuwait during the implementation of covid-19 measures, and having internet access. At the end of the survey period, we collected a total of 520 complete responses, of which 430 participants fulfilled the inclusion criteria. Thus, our final sample size has an estimated power of, approximately, greater than 90%, assuming that the prevalence of psychological disorders is 10% and the margin of error is 5%.

### 2.2 Ethical approval *and consent*

This study was approved by the Kuwait Ministry of Health (MoH) ethics committee (approval number 2020/1470), and all related research activities were performed in accordance with the committee’s guidelines. Parents of the participants received an online information/consent form to read and approve before starting the survey. Parents who signed the consent were allowed to access the study questionnaire. Thus, we confirm that an informed consent from the parent and/or legal guardian for study participation has been obtained from all participants.

### 2.3 Data collection and evaluation tools

We used a structured questionnaire (see **Supplementary Files 1**, **2**) comprising three major domains, including questions about participants’ characteristics, child anxiety using the psychometric properties of the Screen for Child Anxiety Related Emotional Disorders Questionnaire (SCARED; parent version), and child emotions and behaviors using the Strengths and Difficulties Questionnaire (SDQ; parent version). We used a certified academic translator to translate the questionnaire from English to Arabic and made it available to participants in both languages (see **Supplementary Files 1**, **2**). The consistency, comprehensiveness, and clarity of the questionnaire were evaluated by a panel of experts consisting of university faculty, schoolteachers, and child psychiatrists. The Arabic versions of the questionnaires of the SCARED and SDQ rating scales and their morbidity cut-offs have been validated elsewhere (40, 41). The SCARED parent rating scale had an estimated Cronbach alpha reliability coefficient (α) = 0.91, representing a robust internal consistency. Furthermore, the SDQ had an acceptable estimated value for the area under the curve (AUC = 0.84 for the total impact and 0.81 for the total difficulties), representing the ability to distinguish between community and clinical samples. Moreover, we piloted both language versions of the questionnaire to 20 members of the general community to evaluate its length, clarity and consistency. Arabic SCARED and SDQ demonstrated satisfactory psychometric properties in our piloted sample. The participants’ characteristics domain included 25 questions divided into two sections. The first section covered the socio-demographic information for the parents and the child (*e.g.*, age, sex, income, and education). At the same time, the second section consisted of questions related to the status of covid-19 intervention measures (*i.e.*, lockdown status and duration), guardians’ and the child’s past diagnoses of mental illnesses, job status during the pandemic, the child’s screen time behavior, and their coping strategies.

The SCARED rating scale (42) is a child and parent self-report instrument explicitly used to screen for anxiety disorders in children aged between 8 and 18 years old, including general anxiety disorder, separation anxiety disorder, panic disorder and social phobia. In addition, it assesses symptoms related to school phobia. The SCARED consisted of 41 items and 5 factors that parallel the Diagnostic and Statistical Manual of Mental Disorder IV (DSM-IV) classification of anxiety disorders. Each item is rated on a 3-point scale coded as 0 points for hardly ever true, 1 point for somewhat true or sometimes true, and 2 points for very true or often true. The rating scale of all items sums between a minimum of 0 and a maximum of 82 points. In this study, we selected general anxiety disorder as the primary outcome while generalized and social anxieties as the secondary outcomes, based on the relevance of these outcomes to the implemented intervention measures, such as lockdowns and school closures. It is worth noting that general anxiety disorder is defined as the child having any of the broad spectrum of disorders described above, in addition to the normal anxiety that results from everyday stressors. In contrast, generalized anxiety disorder is more specific and defined as a child having excessive paranoia, fear, and tension from any stressors and may require psychiatric intervention (42). However, social anxiety disorder is defined as a child feeling excessively uncomfortable in everyday social situations and may also require an intervention.

We calculated the for each outcome to evaluate their internal consistency. An estimated α between 0.74 and 0.93 for the general scale and the subscale is considered a good internal consistency (42). In this study, we estimated an α = 0.92 for the general scale, while αs = 0.85, and 0.86 for the generalized and social anxieties subscales, respectively. Furthermore, the score of each outcome was dichotomized into 0 and 1 based on the indicated cut-offs of the general scale (*i.e.*, a total score of ≥ 25 indicates the presence of an anxiety disorder) and subscales (*i.e.*, a cutoff of 9 and 8 indicates generalized and social disorders, respectively) for the subsequent statistical analysis.

We used the SDQ rating scale to screen different aspects of the child’s behavioral and emotional aspects developed during the pandemic and associated with anxiety disorders (43). SDQ was designed to detect psychological disorders in children between 3 and 16 years old by assessing subscales related to emotional symptoms, prosocial behaviors, conduct problems, hyperactivity/inattention, and peer relationship problems (44). SDQ has a total of 25 items, in which each subscale is evaluated by 5 items. Like the SCARED rating scale described above, each item is rated on a 3-point scale, generating 10 points for each of the five subscales. Because we aimed to estimate the total difficulties score, we excluded the prosocial subscale, resulting in a total score of 40 and an estimated α = 0.80. Then we used the original three-band categorization scheme, which classifies a score between 0-13 as normal (coded as 0), 14-16 as borderline (coded as 1), and 17-40 as abnormal (coded as 2).

### 2.4 Statistical analysis

We conducted all statistical analyses using Stata version 16.0 (45). Our final four outcome variables included the categorical forms of general, social, and generalized anxiety disorders extracted from the SCARED scales and the three-band categorical form converted from the SDQ total difficulties scale, as described above. At the same time, our predictors comprised participants’ demographics and status before and during the pandemic. Thus, we summarized our variables using frequencies and relative frequencies and assessed their univariate relationships with each outcome using Chi-square tests (and Fisher’s exact tests when a variable has a cell count less than 5). We used logistic regression analysis for each of the three selected anxiety disorders and ordinal regression analysis for the SDQ-generated outcome to model our multivariate relationships. We used a backward elimination strategy to choose our final models and assessed the statistical significance of all two-way interactions between the predictors in each model. Also, we evaluated the confounding effect of non-significant variables using the classical 10% change in the estimate method (46). Briefly, the backward elimination procedure starts with a model that includes all the variables (formerly known as the saturated model) and gradually removes the predictor with the least statistical significance (*i.e.*, the largest P-values). Our strategy was based on keeping statistically significant predictors (P-values < 0.05) while maintaining the smallest Akaike Information Criterion (AIC) and accounting for confounding variables in the final model (46). Thus, when removing a nonsignificant variable changes the inferred Odds ratios (ORs) of other significant variables by more than 10%, that variable will be considered a confounder and kept in the model regardless of its statistical significance. Finally, the goodness of the fit of the final logistic regression models was evaluated using the Hosmer-Lemeshow statistic testing (HL test). HL test is commonly used in epidemiology to evaluate how well the data fits the final selected risk model. The null hypothesis of the HL test is that the selected model appropriately fits the observed data, while the alternative hypothesis indicates the model does not appropriately fit the data. Therefore, an inferred p-value greater than 0.05 concludes that the model fits well. Similarly, we used the approximate likelihood-ratio method to validate the proportionality of odds across response categories for the SDQ ordinal regression model. More explicitly, the method evaluates whether the inferred ORs between each pair of outcomes across two responses are the same. Non-significant P-values inferred by the approximate likelihood-ratio test indicate that the proportionality assumption is not violated.

## 3 Results

### 3.1 Participants’ demographics and prevalence of the study outcomes

**Table 1** summarizes the demographic data and their statistical relationships with the study outcomes. We found that participants (*i.e.*, guardians who filled out the questionnaire) were primarily mothers (73.1%) aged between 35 to 44 years old (54.5%) and have 1 to 3 children (65.7%). The participants’ children were mostly aged between 16 and 18 (57.5%), while the proportion of males to females was almost similar (**Table 1**). Additionally, most of the participants had a college education ( 60%), Kuwaiti citizenship (75.2%), and a monthly income of over $6000 (50.8%). During the lockdowns and when filling out the questionnaire, most participants were on paid leave (56.6%), experienced the full curfew measure (80.5%) and a lockdown duration of less than 28 days (36.9%).

**Table 1:** Baseline characteristics of enrolled participants and their univariate statistical relationships with the study outcomes (N = 430).

Characteristic	n (%)	Anxiety Disorder (n = 107; 24.83%)	Generalized Anxiety (n = 69; 16.00%)	Social Anxiety (n = 78; 18.09%)	SDQ^a^ Abnormal (n = 87; 20.19%)
Demographics					
Guardian of the child Father Mother Other	97 (22.51) 315 (73.09) 19 (4.41)	0.025*	0.038*	0.111	0.017*
Number of children 1 - 3 4 - 6 > 6	283 (65.66) 124 (28.77) 24 (5.57)	0.267	0.025*	0.348	0.062
Marital status Married Single	369 (85.61) 62 (14.39)	0.197	0.273	0.526	0.005*
Age of the guardian 16 - 34 35 - 44 45 - 54 > 55	11 (2.54) 235 (54.52) 144 (33.41) 41 (0.93)	0.380	0.360	0.196	0.554
Age of the child 8 - 11 12 - 15 16 - 18	64 (15.55) 116 (26.91) 248 (57.54)	0.831	0.597	0.589	0.119
Sex of the child Female Male	202 (46.87) 229 (53.13)	0.001*	< 0.001*	0.018*	0.435
Father’s education High school or less Collage Post-graduate	58 (13.46) 252 (58.47) 121 (28.07)	0.004*	0.280	0.042*	< 0.001*
Mother’s education High school or less Collage Post-graduate	23 (5.34) 260 (60.32) 148 (34.34)	0.033*	0.203	**0.021**	0.009*
Monthly income (in USD) < 2000 2000 - 3000 4000 - 5000 > 6000	14 (3.25) 74 (17.17) 124 (28.77) 219 (50.81)	0.463	0.004*	0.868	0.041*
Nationality Citizen Resident	324 (75.17) 107 (24.83)	0.252	0.017*	0.445	0.120
Participants and their children’s characteristics during the pandemic					
Type of lockdown Partial Full	84 (19.49) 347 (80.51)	0.147	0.131	0.801	0.045*
Lockdown duration < 28 days 4 - 8 weeks 8 - 12 weeks > 12 weeks	159 (36.89) 100 (23.20) 69 (16.01) 103 (23.90)	0.001*	0.001*	0.002*	0.019*
Job status during the pandemic Less hours Paid leave Regular hour Unpaid leave More hours Lost a job	97 (22.51) 244 (56.61) 29 (6.73) 25 (5.80) 20 (4.64) 16 (3.71)	0.060	< 0.001*	0.002*	< 0.05*
Child stress at home during the pandemic Yes No	248 (57.54) 183 (42.46)	< 0.001*	< 0.001*	0.005*	< 0.001*
Child mixing with covid-19 cases Yes No	14 (3.25) 417 (96.75)	0.353	0.097	0.706	0.454
Practiced coping strategies during the pandemic Yes No	414 (96.06) 17 (3.94)	0.065	0.245	0.060	0.011*
Child’s screen hour prior to pandemic < 2 hours 2 - 3 hours 4 - 5 hours 6 - 8 hours > 8 hours	159 (36.89) 141 (32.71) 76 (17.63) 25 (5.80) 30 (6.96)	0.806	0.595	0.182	0.002*
Child’s screen hours during to pandemic < 2 hours 2 - 3 hours 4 - 5 hours 6 - 8 hours > 8 hours	38 (8.82) 66 (15.31) 107 (24.83) 102 (23.67) 118 (27.38)	0.083	0.011*	0.332	0.275

^*^Significant p-value at 0.05; ^a^Strengths and Difficulties Questionnaire

Significant p-values are boldfaced.

**Figure 1:**
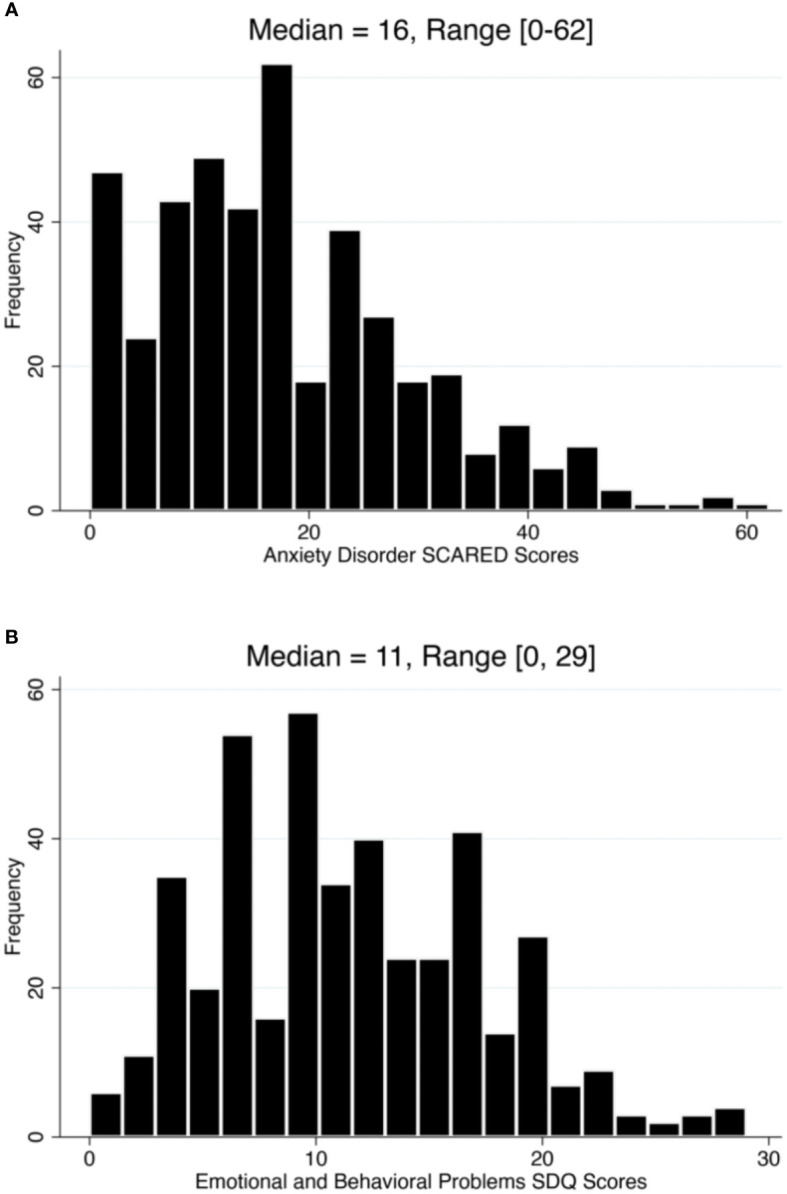
Distribution of anxiety disorders and psychosocial behavioral problems scores among children in the state of Kuwait between April and July 2020. **(A)** histogram showing the score distribution of general anxiety based on the Screen for Child Anxiety-Related Disorders (SCARED) rating scale. **(B)** histogram showing the score distribution of psychosocial behavioral problems based on the Strengths and Difficulties Questionnaire (SDQ) rating scale.

**Figure 2:**
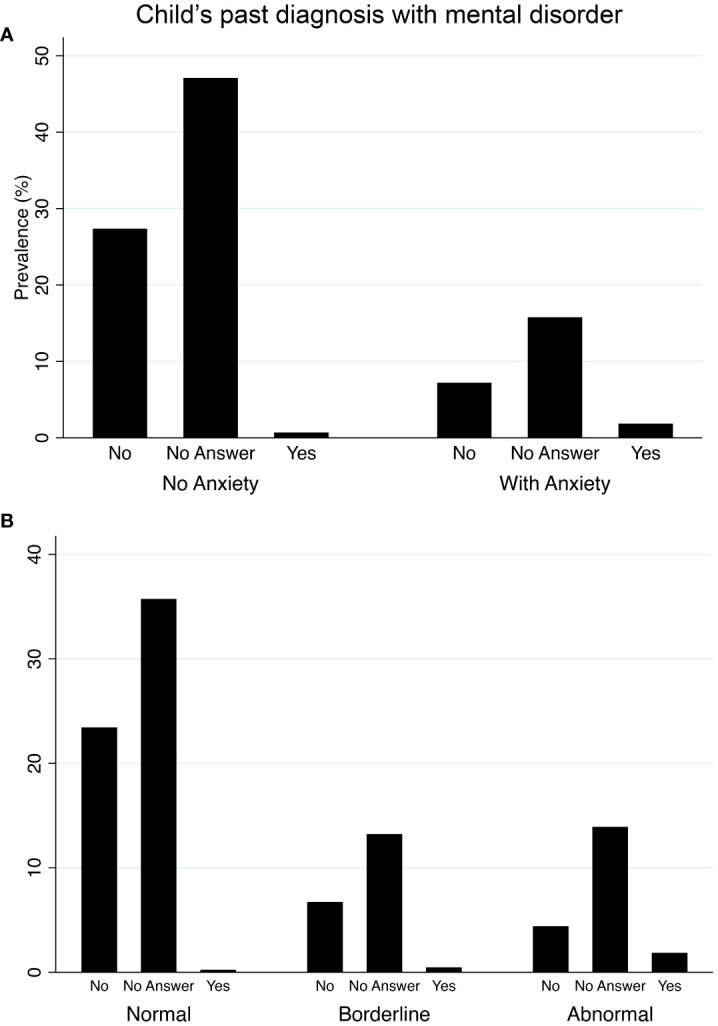
Prevalence of anxiety disorders and psychosocial behavioral problems among children in the state of Kuwait between April and July 2020 in relation to their past diagnosis prior to the pandemic. **(A)** bar chart showing the prevalence of general anxiety based on the Screen for Child Anxiety-Related Disorders (SCARED) rating scale. **(B)** bar chart showing the prevalence of psychosocial behavioral problems based on the Strengths and Difficulties Questionnaire (SDQ) rating scale.

Our median SCARED score was equal to 16 and ranging between 0 and 62 (**Figure 1A**), while the median for the SDQ scores was equal to 11 and ranging between 0 and 29 (**Figure 1B**). We inferred that 24.83% had at least one anxiety disorder. Additionally, results indicate that 16.0% and 18.1% of the children have generalized and social anxiety disorders, respectively (**Table 1**). We found that 20.2% of our participants were classified as abnormal on the SDQ total difficulties scale. Notably, results illustrate that approximately 8% and 15% of the participants either reported no past mental illness or didn’t answer the related question, respectively, whilst their children were diagnosed with an anxiety disorder based on our SCARED rating scale (**Figure 2A**). Similarly, 5% and 15% of the parents either reported no past mental illness or didn’t answer the related question, respectively, but their children were classified as abnormal on the SDQ scale (**Figure 2B**). It is worth noting that 57.5% of the parents reported that their children suffered from stress symptoms during the lockdowns. However, approximately 96% of the children did not mix with active COVID-19 cases, and their parents practiced coping strategies during the lockdowns. Moreover, we found a remarkable increase in the child’s screen time during the lockdowns (*i.e.*, from 7.0% to 27.4% for greater than 8 hours of screen time; **Table 1**) compared to before the pandemic.

### 3.2 Univariate statistical *relationships with the probable* prevalences of study outcomes

Overall, our results suggest significant statistical relationships, inferred using Chi-square tests, between the presence of anxiety disorders (including generalized and social anxieties, as well as behavioral and emotional difficulties) and participants’ demographics, such as the type of child’s guardian, number of children, sex of the child, parents’ education, monthly income, and nationality (p-values < 0.05; **Table 1**). Similarly, we found statistically significant relationships (p-values < 0.05) between the probable prevalence of the study outcomes and children’s characteristics under the pandemic restrictions, including lockdown type and duration, guardian’s job status, child’s stress symptoms, practicing coping strategies, and child’s screen time before the pandemic. Nevertheless, our analysis highlighted the importance of family socioeconomic status and intensity of public health restrictions with the broad-spectrum prevalence of anxiety disorders (**Table 1**). This notion has been reflected through the strongly inferred statistical relationships (P-values < 0.01) with the characteristics of the participants under pandemic restriction including lockdown duration, parents’ job status, and observed symptoms of child stress.

### 3.3 Multivariate associations with child’s anxiety disorders and behavioral and emotional difficulties

Our multivariate analysis (**Table 2**) suggests that the sex of the child (being female) and lockdown durations (increased durations) were consistently significant predictors (ORs > 1; p-values < 0.05) of all anxiety disorders. Further, we found that the sex of the primary guardian (*i.e.*, mainly when the guardian is the mother) was significantly associated with general anxiety disorders in children (OR = 1.90). In Addition, results indicate that guardians’ job status, particularly those who worked for more extended hours or lost their job (OR = 6.0), and children exhibiting apparent signs of stress at home significantly increased the likelihood (ORs > 1; p-values < 0.05) of generalized and social anxieties.

**Table 2:** Multivariate logistic regression model for the risk factors associated with different anxiety disorders.

Risk factor	Odds ratio	95% confidence interval	P-value
Anxiety disorder^a^			
Guardian of the child Father Mother Other	Reference = 1 **1.90** 0.94	**(1.02, 3.57)** (0.23, 3.81)	**0.045** 0.933
Sex of the child Male Female	Reference = 1 **3.01**	**(1.89, 4.75)**	**0.001**
Lockdown duration < 28 days 4 - 8 weeks 8 - 12 weeks > 12 weeks	Reference = 1 1.49 **3.02** **3.24**	(0.77, 2.86) **(1.54, 5.90)** (**1.76, 5.97)**	0.230 **0.001** **< 0.001**
Generalized Anxiety disorder^b^			
Sex of the child Male Female	Reference = 1 **2.5**	**(1.4, 5.47)**	**< 0.001**
Lockdown duration < 28 days 4 - 8 weeks 8 - 12 weeks > 12 weeks	Reference = 1 **2.63** **3.32** **7.14**	**(1.10, 6.30)** **(1.32, 8.31)** **(3.15, 16.21)**	**0.029** **0.010** **< 0.001**
Job status during the pandemic Less hours Paid leave Regular hour Unpaid leave More hours Lost a job	Reference = 1 0.85 **0.11** 1.09 **4.04** **6.00**	(0.41, 1.78) **(0.01, 0.90)** (0.32, 3.73) **(1.21, 13.45)** **(1.71, 21.08)**	0.678 **0.040** 0.884 **0.023** **0.005**
Child stress at home during the pandemic No Yes	Reference = 1 2.98	(1.52, 5.81)	0.001
Social Anxiety disorder^c^			
Sex of the child Male Female	Reference = 1 **2.11**	**(1.26, 7.98)**	**0.021**
Lockdown duration < 28 days 4 - 8 weeks 8 - 12 weeks > 12 weeks	Reference = 1 **3.67** **3.46** **4.33**	**(1.70, 7.94)** **(1.50, 7.96)** **(2.04, 9.18)**	**0.001** **0.003** **< 0.001**
Job status during the pandemic Less hours Paid leave Regular hour Unpaid leave More hours Lost a job	Reference = 1 **2.95** 1.28 **3.58** 2.60 **1.75**	**(1.31, 6.64)** (0.34, 4.81) **(1.05, 12.15)** (0.66, 10.30) **(3.21, 42.98)**	**0.009** 0.713 **0.041** 0.173 **< 0.001**
Child stress at home during the pandemic No Yes	Reference = 1 1.99	(1.13, 3.54)	0.018

Hosmer – Lemeshow goodness-of-fit p-values = 0.14^a^, 0.17^b^, 0.16^c^

Significant ORs (95% CI) and P-values are boldfaced.

Similarly, our ordinal regression results suggest that the guardian of the child, lockdown duration and the guardian’s job status during the pandemic were significant predictors of SDQ three-band categories (**Table 3**). We inferred a remarkable increase in the likelihood of abnormal behavioral and emotional difficulties (OR = 2.25) when the child’s guardian is the mother, as well as when the lockdown duration exceeds 8 weeks (ORs > 2.0). Also, we found notably high associations with abnormal behavioral and emotional difficulties (ORs > 4) when the guardian lost their job or worked for longer hours during the restrictions (**Table 3**).

**Table 3:** Multivariate ordinal logistic regression model for potential risk factors associated with SDQ original categories.

Risk factor	Proportional ORs	95% confidence interval	P-value
Guardian of the child Father Mother Other	Reference = 1 **2.25** 1.58	(1.34, 3.78) (0.52, 4.77)	0.002 0.412
Lockdown duration < 28 days 4 - 8 weeks 8 - 12 weeks > 12 weeks	Reference = 1 1.65 **2.33** **2.40**	(0.98, 2.79) **(1.35, 4.27)** **(1.40, 4.27)**	0.057 **0.003** **0.001**
Job status during the pandemic Less hours Paid leave Regular hour Unpaid leave More hours Lost a job	Reference = 1 1.37 1.18 1.22 **4.15** **4.85**	(0.83, 2.28) (0.50, 2.76) (0.48, 3.08) **(1.64, 10.47)** **(1.66, 14.20)**	0.214 0.702 0.664 **0.003** **0.004**
**Model Intercepts** Normal *vs.* Borderline Borderline *vs.* Abnormal	1.81 2.87	(1.15, 2.46) (2.18, 3.56)	

Approximate likelihood-ratio test of proportionality of odds across response categories, p-value = 0.6. Significant ORs (95% CI) and p-values are boldfaced

## 4 Discussion

We used a cross-sectional study with two popular psychometric assessment tools to uncover the impacts of strict public health measures on children’s mental health, such as lockdowns and school closures. To the authors’ knowledge, this is the first study investigating the prevalence of different anxiety disorders and behavioral and emotional problems among children and young adults in Kuwait during COVID-19 lockdown measures. Here, we found notably significant associations between the prevalence of anxiety disorders, socioeconomic factors, and lockdown duration. These findings are not only critical for shedding epidemiological insights into children’s psychological status during COVID-19 restrictions but also assist with the establishment of intervention programs against the long-term implications of such measures on the children’s mental and physical health.

Our results revealed a remarkably high probable prevalence of anxiety disorders and psychosocial behavioral problems among children aged between 8 and 18 in Kuwait (≥ 20%; **Table 1**). Our inferred prevalence of mental disorders was similar to past studies with larger samples from different populations (4, 47, 48). This is not surprising since social isolation and negative information from mainstream media, particularly during emergencies, were significantly associated with psychological disorders (49–51). However, our estimates did not substantially surpass what was estimated before the pandemic in the region, which ranged between approximately 5% and 20% (39, 52). Yet, the Middle East continues to suffer from regional political conflicts and wars before and after the pandemic. Therefore, a comparative epidemiological analysis between the two periods is difficult to achieve, mainly when the population is consistently susceptible to environmental stressors and mental trauma (36, 38). That said, our estimated prevalence of anxieties in Kuwait is considered high and was significantly associated with the pandemic’s characteristics, as described earlier.

Like past studies (16, 53–55), the sex of the child was a significant predictor of anxiety disorders (**Table 2**), in which their prevalence was substantially high in females. In fact, we found that female children are more likely to develop anxiety disorders than males (ORs > 2; **Table 2**). This aggravated prevalence of female anxieties during the pandemic has been attributed to the Middle Eastern sociocultural norms, school closures which led to their social isolation, and exacerbated fear of losing their loved ones (53, 56). In fact, Middle Eastern sociocultural norms have been found to be a major cause of stigma toward mentally ill individuals and have acted as a barrier to seeking care or medical intervention (57). Indeed a remarkable percentage of the participants in this study either claimed no history of mental illness or did not answer the question, while their children were diagnosed with anxiety disorders using our rating scales (**Figure 2**). Thus, our result affirms the importance of increasing public mental health awareness and psychoeducation programs in the region, particularly during emergencies (58).

Nevertheless, it is worth noting that in this study, mothers reported substantially higher anxieties and behavioral problems in their children during lockdowns (**Tables 1**, **3**). Furthermore, our results inferred that mothers are significantly more likely (ORs > 1; p-values < 0.05) to report mental disorders in their children than other guardians (**Tables 2**, **3**). These findings are consistent with the notion that mothers’ parenting distress could mediate additional mental disorders in their children, especially during emergencies (59). This might be attributed to maternal protective instincts, associated recall biases, and the additional burden of homeschooling. In fact, we found that most of these mothers had a college degree, which was significantly associated with them reporting mental disorders in their children (**Table 1**). Such participants are more likely to be working mothers, which adds an additional burden and distress to their parenting role during lockdowns (60).

While most of the guardians were on paid leave during the period of the study, we found significant relationships between children’s mental disorders and their job status (**Tables 1**, **3**). Also, job status was a significant predictor of generalized and social anxiety, psychosocial, and behavioral problems (**Tables 2**, **3**). However, our multivariate analysis revealed that guardians who worked regular hours, their children are less likely to have generalized anxiety (OR = 0.11; p-value = 0.040; **Table 2**). This result might be attributed to the fact that the interaction between parents and children during the day is on a normal basis, and therefore, children are less likely to experience stressful negative encounters with their parents. In contrast, we found that guardians with paid leave and their children are more likely to have social anxiety (OR = 2.95; p-value = 0.009; **Table 2**). This can be either attributed to the parent being very protective of their children to keep them safe, hindering the development of their social skills, or the indirect impacts of school closures (56, 61). Despite the small number of guardians who lost their jobs due to the pandemic restrictions (**Table 1**), our multivariate analysis consistently showed that such economic implications substantially increase the likelihood (ORs > 1; p-value < 0.05; **Tables 2**, **3**) of children having psychological disorders. Indeed, job loss, in addition to pandemic stress, was shown to have devastating impacts on the parent’s mental health, which subsequently reflects on their children (62). Further, children with guardians working extra hours during the pandemic were more likely to have generalized anxiety and psychosocial behavioral problems due to the abnormal lack of parenting activities (**Tables 2**, **3**). Also, one would consider the notion that the age of the child is potentially a significant predictor or a confounder over this relationship between child anxiety and the duration of parental social interactions due to job status. However, the result of our study consistently inferred that the age of the child has neither a significant statistical relationship (**Table 1**) nor a predictor (**Tables 2**, **3**) with the study outcomes, as suggested elsewhere (59). This might be attributed to the fact that the age range (*i.e.*, between 8 and 18 years) does not sufficiently confound individual susceptibility to mental health disorders by being within that age group.

We found that the type of lockdown (*i.e.*, full or partial) was not a significant predictor of psychological disorders in children. Yet, the lockdown duration was consistently a significant predictor of anxiety and psychosocial, behavioral disorders (**Tables 2**, **3**). Additionally, we inferred notable positive correlations in the ORs as the duration of the lockdowns increased, resembling a dose-response relationship (**Tables 2**, **3**). Kuwait imposed a full lockdown on the 10^th^ of May 2020 for three weeks, followed by prolonged irregular partial lockdowns till the 22^nd^ of April 2021. These findings are consistent with past studies on estimating the increased longitudinal trajectories of the prevalence of anxiety and depression among children and adults from such prolonged public health restrictions (8, 63). These studies also showed various outcomes among affected individuals from different risk groups (*e.g.*, the autistic population), including the worsening of their existing psychological disorders and the development of additional new mental disorders (5, 9).

Our study inferred a significantly high probable prevalence of psychological disorders among children from low-income families (**Table 1**). This indicates that the additive combination of prolonged school closures and low socioeconomic status constitute the highest risk factors for observing an increased prevalence of mental disorders among children during emergencies. This is because schools generally provide appropriate environments for childcare and enrichment activities, which are lacking in most low-income families (64). Additionally, such implications might also affect poorly performing students from all socioeconomic strata, primarily due to the prolonged school closures and the limitations of online education (53, 65). Although many families in our sample practiced coping strategies (**Table 1**), we could not show consistent positive or protective effects that lower the prevalence of psychological disorders. This is expected since this is a cross-sectional study, and there were no public health awareness campaigns or education programs for coping with such emergencies, particularly during the first year of the pandemic. Yet, such circumstances and related consequences on children’s mental health were similar elsewhere, on the levels of the Middle East (34, 36, 53) and the worldwide (4, 5). Finally, while children’s screen hours during the pandemic were not a significant predictor of mental disorders, we could infer significantly fewer hours prior to the pandemic in relation to psychosocial problems (**Table 2**). Also, our results suggested significantly more screen hours during the pandemic in relation to generalized anxiety (**Table 1**). These findings are another indication of the potential negative implications of school closures that, in particular, exacerbate the use of social media, leading to the further progression of children’s mental disorders (28). Therefore, an intervention is needed to mitigate such behavioral problems on the level of the parents as well as the educational and public health authorities.

The first limitation of the present study is that our findings were based on a cross-sectional study in which causal relationships cannot be inferred. Yet, our conclusions regarding the significant associations between the prevalence of mental disorders in children and lockdowns were psychologically plausible and strongly agreed with the published literature described above. Second, the use of convenience sampling through social media platforms may hinder the generalizability of the results to the target population due to selection bias. However, our sample was able to cover an acceptable proportionality in terms of sex and age of the children, as well as, socioeconomic strata in the state of Kuwait (**Table 1**). Therefore, in addition to the plausibility of the inferred results, our results may have some representativeness to the target population. Moreover, our data were derived from a self-reported survey by the guardians rather than a face-to-face interview, which might have had more validity. Additionally, self-reporting questionnaires suffer from high rates of recall bias. Finally, the present study does not sufficiently explore the relationship between parental bonding (*e.g.*, reflected by equally sharing the responsibility of the childcare) and the development of anxiety disorders in children. As past studies found, poor quality bonding between the parents, as well as with their children, are strongly significant predictors of short and long-term development of anxiety and other mental disorders in children up to their late adulthood (66, 67). Although we attempted this by assessing the statistical significance of the interaction between the type of child guardian and marital status, we could not detect such significance. This might be attributed to most mothers and fathers being married (*i.e.*, 86.0% and 94%, respectively), while a small proportion (*i.e.*, 14.0% and 5.2%, respectively) are single. Furthermore, a more in-depth section in the questionnaire investigating parenthood quality was needed, as described elsewhere (66, 67). Thus, future studies should consider avoiding these limitations and estimating mental disorder incidences and trends, especially after school re-openings.

## 5 Conclusions

The study represents the first attempt to assess the epidemiological status of mental health disorders in children during the first year of the pandemic when school closures and lockdowns were implemented in Kuwait. We found a remarkably high probable prevalence of anxiety disorders and psychosocial, behavioral problems among children aged between 8 and 18, especially among females. Our most important finding was that lockdown duration was consistently a significant predictor of the broad spectrum of selected mental disorders. Additionally, we inferred notable increases in the likelihood of mental disorders as the duration of the lockdowns increased. Moreover, lockdowns, in combination with school closures, might be associated with the increased prevalence of such disorders, particularly in children belonging to families with lower socioeconomic statuses. Our findings revealed preliminary insights into the vulnerability of young populations to the indirect negative impacts of strict public health measures during pandemic emergencies. Thus, authorities should consider such implications when planning and implementing similar interventions in future pandemics. Also, targeted mental-health surveillance programs for high risk-groups must be initiated to assess and prevent the long-term implications associated with post-pandemic psychological trauma. Therefore, massive and innovative resources must be invested in improving children’s coping abilities, particularly during school re-openings.

## Data availability statement

The raw data supporting the conclusions of this article will be made available by the authors, without undue reservation.

## Ethics statement

The studies involving humans were approved by Kuwait Ministry of Health (MoH) ethics committee. The studies were conducted in accordance with the local legislation and institutional requirements. Written informed consent for participation in this study was provided by the participants’ legal guardians/next of kin.

## Author contributions

BA: Conceptualization, Funding acquisition, Investigation, Methodology, Project administration, Resources, Supervision, Writing – original draft, Writing – review & editing. MAA: Conceptualization, Data curation, Formal analysis, Investigation, Methodology, Software, Validation, Visualization, Writing – original draft, Writing – review & editing. AN: Data curation, Investigation, Writing – review & editing. HA: Data curation, Investigation, Writing – review & editing. MAS: Investigation, Validation, Writing – original draft, Writing – review & editing.

## Supplementary material

Supplementary File 1 contains the English version of the questionnaire.

Supplementary File 2 contains the Arabic version of the questionnaire.
