# Supplementary material for: Anxiety disorders among children and adolescents during COVID-19 lockdowns and school closures: a cross-sectional study in Kuwait
Source: Front Psychiatry. 2024 Feb 12;15:1322745. doi: 10.3389/fpsyt.2024.1322745 (PMC10895000; doi:10.3389/fpsyt.2024.1322745)
Supplement: Supplementary File 1 — contains the English version of the questionnaire. [file DataSheet_1.docx]

Anxiety Disorders among Children and Adolescents during COVID-19 Lockdowns and School Closures: A Cross-Sectional Study in Kuwait

Bibi Alamiri^1☯^, Moh A. Alkhamis^2*☯^, Ahmed Naguy^1^, Hend F Alenezi^3^, Muna Al Shekaili^4^

* Required

معلومات تخص معبء الاستبانة

In this section we will ask you few questions about yourself

أنا *

والد الطفل /ة

والدة الطفل/ة

Other:

كم طفل لديك *

1-3

4-6

أكثر من 6

الحالة الاجتماعية *

متزوج/ة

منفصل/ة

مطلق/ة

أرمل/ة

المستوى التعليمي للأب *

لا يقرأ ولا يكتب

يستطيع القراءة والكتابة

المستوى الاعدادي

الدبلوم العام

باكلوريوس

دراسات عليا

المستوى التعليمي للأم *

لا يقرأ ولا يكتب

يستطيع القراءة والكتابة

المستوى الاعدادي

الدبلوم العام

باكلوريوس

دراسات عليا

دخل العائلة الشهري (دينار كويتي)*

أقل من ٥٠٠

٥٠٠-١٠٠٠

١٠٠٠- ١٥٠٠

اكثر من ١٥٠٠

الجنسية *

عماني

سعودي

قطري

كويتي

بحريني

اماراتي

Other:

خلال فترة الجائحة نحن متواجدون في *

سلطنة عمان

المملكة العربية السعودية

دولة قطر

دولة الكويت

دولة البحرين

دولة الامارات العربية المتحدة

هل تم تشخيصك في السابق بأي مرض نفسي *

نعم

لا

أفضل عدم الاجابة

أذا كانت الاجابة نعم ما هو تشخيصك *

لا يوجد تشخيص سابق

الاكتئاب

اضطراب الوسواس القهري

اضطرابات النوم

اضطراب ثنائي القطبي

الذهان

اضطراب طيف التوحد

اضطراب فرط الحركة وتشتت الانتباه

تخلف عقلي بسيط

تخلف عقلي متوسط الى شديد

Other:

معلومات الطفل

اختر احد أطفالك الذي يتراوح اعمارهم بين عمر 8 سنوات الى 18 سنة وأجب على الاسئلة التالية

عمر الطفل *

اقل من 8 سنوات

8-11 سنة

12-15 سنة

16-18 سنة

جنس الطفل *

ذكر

أنثى

المستوى التعليمي للطفل *

قبل المدرسة

صف الاول - الصف الثاني

الصف الثالث- الصف السادس

الصف السابع الى الصف العاشر

الصف 11 الى الصف 12

Other:

هل شعر ابنك بالضغط النفسي منذ بداية الجائحة *

نعم

لا

هل تعامل ابنك مع حالة تم تشخيصها بكورونا *

نعم

لا

ما طبيعة تعامل طفلك مع حالة كورونا *

لم يتعامل

لقد أصيب/ت بمرض فايروس كورونا

لقد أصيب احد والديه بفايروس كورونا

أصيب أحد أخوانه

أصيب أحد الاقارب

أصيب أحد أصدقاؤه

ماذا كانت حدة الحالات التي تعامل معها طفلك *

لم يتعامل

الحالة كانت خفيفة (لم تتطلب عناية طبية)

الحالة كانت متوسطة (تنويم في المستشفى )

شديدة (تنويم في وحدة العناية المركزة )

حدثت وفاة

هل تم تشخيص طفلك/تك سابقا باي من الامراض النفسية *

نعم

لا

أفضل عدم الافصاح

أذا كانت الاجابة نعم ما هو تشخيص طفلك/تك *

لا يوجد تشخيص سابق

الاكتئاب

اضطراب الوسواس القهري

اضطرابات النوم

اضطراب ثنائي القطبي

الذهان

اضطراب طيف التوحد

اضطراب فرط الحركة وتشتت الانتباه

تخلف عقلي بسيط

تخلف عقلي متوسط الى شديد

Other:

مقياس اختبار الاضطرابات المتعلقة بالقلق عند الاطفال (للوالدين )

ا
الارشادات: أدناه لائحة عبارات تصف كيفية شعور الناس.
اقرا كل جملة و قرر اذا كانت " غير صحيحة أو بالكاد صحيحة"، أو " صحيحة غالبا عن ولدك
وهذه الاسئلة الذي و يصف
ولدك خلال الاشهر الثلاث الماضٌية. رجاء" حاول ان تجاوب على كل البنود بأنسب طرٌيقة، حتى لو أن بعض الجمل لا تعنًي ولدك

1.عندما يشعر ولدي بالخوف، ٌيصعب عليه التنفس *

غير صحيح او بالكاد صحيح

صحيح نوع ما أو صحيح احيانا

صحيح , صحيح غالبا

2- يصاب ولدي بأالم في الرأس عندما يكون في المدرسة *

غير صحيح او بالكاد صحيح

صحيح نوع ما أو صحيح احيانا

صحيح , صحيح غالبا

3-لا يحب ولدي أن يتواجد مع أشخاص لا يعرفهم جيدا . *

غير صحيح او بالكاد صحيح

صحيح نوع ما أو صحيح احيانا

صحيح , صحيح غالبا

4- يشعر ولدي بالخوف اذا نام خارج المنزل. *

غير صحيح او بالكاد صحيح

صحيح نوع ما أو صحيح احيانا

صحيح , صحيح غالبا

5- يقلق ولدي من أن لا يحبه الاخرون *

غير صحيح او بالكاد صحيح

صحيح نوع ما أو صحيح احيانا

صحيح , صحيح غالبا

6-عندما يشعر ولدي بالخوف، يشعر و كأنه سيغمى عليه. *

غير صحيح او بالكاد صحيح

صحيح نوع ما أو صحيح احيانا

صحيح , صحيح غالبا

7- ولدي متوتر. *

غير صحيح او بالكاد صحيح

صحيح نوع ما أو صحيح احيانا

صحيح , صحيح غالبا

8- ولدي يتبعني حيثما أذهب *

غير صحيح او بالكاد صحيح

صحيح نوع ما أو صحيح احيانا

صحيح , صحيح غالبا

9- يقول لي الناس ان ولدي يبدو متوتر *

غير صحيح او بالكاد صحيح

صحيح نوع ما أو صحيح احيانا

صحيح , صحيح غالبا

10- يشعر ولدي بالتوتر برفقة أشخاص لا يعرفهم جيدا" *

غير صحيح او بالكاد صحيح

صحيح نوع ما أو صحيح احيانا

صحيح , صحيح غالبا

11- .يصاب ولدي بألم في المعدة عندما يكون في المدرسة *

غير صحيح او بالكاد صحيح

صحيح نوع ما أو صحيح احيانا

صحيح , صحيح غالبا

12- عندما يشعر ولدي بالخوف، يشعر و كأنه يفقد صوابه *

غير صحيح او بالكاد صحيح

صحيح نوع ما أو صحيح احيانا

صحيح , صحيح غالبا

13- يقلق ولدي من النوم لوحده. *

غير صحيح او بالكاد صحيح

صحيح نوع ما أو صحيح احيانا

صحيح , صحيح غالبا

14- يقلق ولدي من كونه أقل جدارة من الاولاد االاخرين. *

غير صحيح او بالكاد صحيح

صحيح نوع ما أو صحيح احيانا

صحيح , صحيح غالبا

15- عندما يشعر ولدي بالخوف، يشعر بأن الاشياء غير حقيقية *

غير صحيح او بالكاد صحيح

صحيح نوع ما أو صحيح احيانا

صحيح , صحيح غالبا

16- تنتاب ولدي الكوابيس من اصابة والديه بمكروه *

غير صحيح او بالكاد صحيح

صحيح نوع ما أو صحيح احيانا

صحيح , صحيح غالبا

17- يقلق ولدي من الذهاب الى المدرسة *

غير صحيح او بالكاد صحيح

صحيح نوع ما أو صحيح احيانا

صحيح , صحيح غالبا

18- عندما يشعر ولدي بالخوف ، يخفق قلبه بسرعة. *

غير صحيح او بالكاد صحيح

صحيح نوع ما أو صحيح احيانا

صحيح , صحيح غالبا

19- يرتجف ولدي *

غير صحيح او بالكاد صحيح

صحيح نوع ما أو صحيح احيانا

صحيح , صحيح غالبا

20- تنتاب ولدي كوابيس أن يتعرض لمكروه *

غير صحيح او بالكاد صحيح

صحيح نوع ما أو صحيح احيانا

صحيح , صحيح غالبا

21- يقلق ولدي من أن تتعرقل أموره *

غير صحيح او بالكاد صحيح

صحيح نوع ما أو صحيح احيانا

صحيح , صحيح غالبا

22- عندما يشعر ولدي بالخوف،يتعرق كثيرا" *

غير صحيح او بالكاد صحيح

صحيح نوع ما أو صحيح احيانا

صحيح , صحيح غالبا

23- ولدي قلق *

غير صحيح او بالكاد صحيح

صحيح نوع ما أو صحيح احيانا

صحيح , صحيح غالبا

24- يشعر ولدي بالخوف الشدٌيد بدون سبب اطلاقا" *

غير صحيح او بالكاد صحيح

صحيح نوع ما أو صحيح احيانا

صحيح , صحيح غالبا

25- يخاف ولدي من أن يبقى لوحده في المنزل *

غير صحيح او بالكاد صحيح

صحيح نوع ما أو صحيح احيانا

صحيح , صحيح غالبا

26- يصعب على ولدي التحدث مع أشخاص لا يعرفهم جيدا *

غير صحيح او بالكاد صحيح

صحيح نوع ما أو صحيح احيانا

صحيح , صحيح غالبا

27- عندما يشعر ولدي بالخوف، يشعر و كأنه يختنق. *

غير صحيح او بالكاد صحيح

صحيح نوع ما أو صحيح احيانا

صحيح , صحيح غالبا

28- يقول لي الناس ان ولدي ٌقلق كثٌرا". *

غير صحيح او بالكاد صحيح

صحيح نوع ما أو صحيح احيانا

صحيح , صحيح غالبا

29- لا يحب ولدي أن يكون بعٌيدا عن عائلته. *

غير صحيح او بالكاد صحيح

صحيح نوع ما أو صحيح احيانا

صحيح , صحيح غالبا

30- يخاف ولدي من أن تصيبه نوبات قلق أو هلع. *

غير صحيح او بالكاد صحيح

صحيح نوع ما أو صحيح احيانا

صحيح , صحيح غالبا

31- يقلق ولدي من أن يحصل مكروه لوالديه. *

غير صحيح او بالكاد صحيح

صحيح نوع ما أو صحيح احيانا

صحيح , صحيح غالبا

32- يشعر ولدي بالخجل مع الاشخاص الذين لا يعرفهم جيدا . *

غير صحيح او بالكاد صحيح

صحيح نوع ما أو صحيح احيانا

صحيح , صحيح غالبا

33- يقلق ولدي مّما سيحصل في المستقبل *

غير صحيح او بالكاد صحيح

صحيح نوع ما أو صحيح احيانا

صحيح , صحيح غالبا

34- عندما يخاف ولدي يشعر بأنه سوف يتقيأ. *

غير صحيح او بالكاد صحيح

صحيح نوع ما أو صحيح احيانا

صحيح , صحيح غالبا

35- يقلق ولدي من أن لا يقوم بالاشياء بشكل جيد *

غير صحيح او بالكاد صحيح

صحيح نوع ما أو صحيح احيانا

صحيح , صحيح غالبا

36- يخاف ولدي من الذهاب الى المدرسة *

غير صحيح او بالكاد صحيح

صحيح نوع ما أو صحيح احيانا

صحيح , صحيح غالبا

37- تقلق ولدي أمورقد حصلت. *

غير صحيح او بالكاد صحيح

صحيح نوع ما أو صحيح احيانا

صحيح , صحيح غالبا

38- عندما يشعر ولدي بالخوف، يشعر بالدوار *

غير صحيح او بالكاد صحيح

صحيح نوع ما أو صحيح احيانا

صحيح , صحيح غالبا

39- يشعر ولدي بالتوتر عندما ٌكون برفقة االاولاد أوالكبارالاخرين وعليه القيام بشيء بينما يراقبونه (مثلا القراءة بصوت عالي , التكلم , اللعب , القيام بالرياضة ) *

غير صحيح او بالكاد صحيح

صحيح نوع ما أو صحيح احيانا

صحيح , صحيح غالبا

40- .يشعر ولدي بالتو ّتر عندما يذهب الى حفلات أو أيه مكان يتواجد فيه أشخاص لا يعرفهم جيدا". *

غير صحيح او بالكاد صحيح

صحيح نوع ما أو صحيح احيانا

صحيح , صحيح غالبا

41- ولدي خجول *

غير صحيح او بالكاد صحيح

صحيح نوع ما أو صحيح احيانا

صحيح , صحيح غالبا

أستبيان مواطن القوة والصعوبة

يرجى الاجابة على كل بند ب : غير صحيح, صحيح نوعا ما , او صحيح بالتاكيد
حاول ان تكون دقيقا في اجابتك سوف يساعدنا كثيرا اذا اجبت على كل بند حتى وان كنت غير متاكد او ترى انه غير مناسب.
يرجى ان تكون اجابتك حول سلوك الطفل خلال الستة الأشهر الأخير

يهتم بمشاعر الاخرين *

غير صحيح

صحيح نوعا ما

صحيح بالتاكيد

2- لا يستطيع البقاء او الاستقرار فى مكان واحد . كثير الحركة *

غير صحيح

صحيح نوعا ما

صحيح بالتاكيد

3- كثيرا ما يشكو من صداع او آلام فى البطن او الشعور بالغثيان *

غير صحيح

صحيح نوعا ما

صحيح بالتاكيد

4- يشرك الاخرين بسهولة فيما يخصه{ لعب, أقلام, ألعاب, حلويات .....الخ) *

غير صحيح

صحيح نوعا ما

صحيح بالتاكيد

5-كثيرا ما تنتابة نوبات من الغضب الشديد أ و سريع الغضب *

غير صحيح

صحيح نوعا ما

صحيح بالتاكيد

6-يحب العزلة. يميل الى اللعب لوحدة *

غير صحيح

صحيح نوعا ما

صحيح بالتاكيد

7- مطيع على وجه العموم. عادة يفعل ما يطلبه منه الكبار *

غير صحيح

صحيح نوعا ما

صحيح بالتاكيد

8-يقلق من اشياء كثيرة. كثيرا ما يبدو عليه القلق *

غير صحيح

صحيح نوعا ما

صحيح بالتاكيد

9-يساعد الاخرين اذا ما حدث لأحدهم مكروه *

غير صحيح

صحيح نوعا ما

صحيح بالتاكيد

10-يتململ او يتلوى باستمرار{جسمه فى حركه مستمره اثناء جلوسه) *

غير صحيح

صحيح نوعا ما

صحيح بالتاكيد

11-لديه على الاقل صديق واحد جيد *

غير صحيح

صحيح نوعا ما

صحيح بالتاكيد

12-كثيرا ما يتعارك مع الاخرين من نفس سنه او يستأسد عليهم *

غير صحيح

صحيح نوعا ما

صحيح بالتاكيد

13-كثيرا ما يكون غير سعيد, حزين او يبكى بسهوله *

غير صحيح

صحيح نوعا ما

صحيح بالتاكيد

14-فى الغالب محبوب ممن هم فى سنه *

غير صحيح

صحيح نوعا ما

صحيح بالتاكيد

15-يتشتت انتباهه بسرعه وقليل التركيز *

غير صحيح

صحيح نوعا ما

صحيح بالتاكيد

16-عصبى او متشبث{متعلق} بالاخرين فى المواقف الجديدة. من السهل ان يفقد ثقته بنفسه *

غير صحيح

صحيح نوعا ما

صحيح بالتاكيد

17-لطيف مع من هم أصغر منه *

غير صحيح

صحيح نوعا ما

صحيح بالتاكيد

18- كثيرا ما يكذب , يخدع او يغش *

غير صحيح

صحيح نوعا ما

صحيح بالتاكيد

19-يستهزأ ْ منه او يستاسد عليه من هم فى سنه *

غير صحيح

صحيح نوعا ما

صحيح بالتاكيد

20- كثيرا ما يتطوع لمساعدة الاخرين{الوالدين, المدرسين, الاطفال الاخرينْ *

غير صحيح

صحيح نوعا ما

صحيح بالتاكيد

21-يفكر قبل ان يتصرف *

غير صحيح

صحيح نوعا ما

صحيح بالتاكيد

22-يسرق من البيت او المدرسة او من أماكن أخرى *

غير صحيح

صحيح نوعا ما

صحيح بالتاكيد

23-ينسجم بشكل مع الكبار عنه مع الاطفال فى نفس سنه *

غير صحيح

صحيح نوعا ما

صحيح بالتاكيد

24- يخاف من اشياء كثيره . من السهل تخويفه *

غير صحيح

صحيح نوعا ما

صحيح بالتاكيد

25- يتابع اداء الواجبات حتى النهايه. لديه انتباه جيد *

غير صحيح

صحيح نوعا ما

صحيح بالتاكيد

بصفة عامة هل تعتقد ان لدى الطفل صعوبات فى مجال واحد او أكثر من المجالات الآتية.الأنفعالات اوالعواطف , التركيز, السلوك, او انسجامه وتعامله مع الآخرين ؟ *

لا

نعم صعوبات بسيطة

نعم صعوبات واضحة

نعم صعوبات شديدة

اذا كانت الأجابة " نعم"؛ أرجو الأجابة على الأسئلة التالية عن هذه الصعوبات:

منذ متى بدأت هذه الصعوبات

أقل من شهر

1-5 أشهر

6-12 شهر

أكثر من سنة

هل يتألم الطفل بسبب هذه الصعوبات

لا

قليلا

متوسطا

كثيرا

هل هذه الصعوبات تؤثر على حياة الطفل اليومية بشكل سلبى فى المجالات التالية :

لا قليلا متوسط كثيرا

Row 1

الحياة المنزلية

علاقته بالاصدقاء

الدراسة

النشاطات الترفيهية

Row 1

الحياة المنزلية

علاقته بالاصدقاء

الدراسة

النشاطات الترفيهية

هل هذه الصعوبات تضع عبئا عليك او على الأسرة ككل

لا

قليلا

متوسط

كثيرا

من بداية الجائحة هل مارست أو اتبعت احد الارشادات التالية

كعائلة نقوم بالعديد من الانشطة الدينية أكثر من المعتاد

طفلي يمارس الرياضات البدنية اكثر

طفلي يمارس التأمل أكثر

طفلي حريص على التةاصل مع اصدقاؤه واهله (عبر الانترنت)

يقلل من متابعة أخبار الجائحة

لم يمارس اي شيء

شكرا لمشاركتك الرجاء الضغط على تسليم "
