# Supplementary material for: Anxiety disorders among children and adolescents during COVID-19 lockdowns and school closures: a cross-sectional study in Kuwait
Source: Front Psychiatry. 2024 Feb 12;15:1322745. doi: 10.3389/fpsyt.2024.1322745 (PMC10895000; doi:10.3389/fpsyt.2024.1322745)
Supplement: Supplementary File 2 — contains the Arabic version of the questionnaire. [file DataSheet_2.docx]

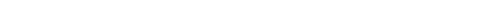


Anxiety Disorders among Children and Adolescents during COVID-19 Lockdowns and School Closures: A Cross-Sectional Study in Kuwait

Bibi Alamiri^1☯^, Moh A. Alkhamis^2*☯^, Ahmed Naguy^1^, Hend F Alenezi^3^, Muna Al Shekaili^4^

**Guardian information**

**Do you have children between the ages of 8 to 18 years?**

Yes

No

**I am the …**

Father of the child

Mother of the child

Other

**How many children do you have** 7.7%

1-3
4-6
more than 6

**Marital status**

Married

Separated

Divorced

Widow

Never married but primary caregiver of a child (e.g. adoptive parents, family members etc.)

**Age**

16-24

25-34

35-44 8.3%

45-54
55-64
65 and older

52.1%

**Education level of the Father**43.8%

illiterate
Can read and write
Primary school
middle school/secondary school college degree
Post-graduate degree

**Education level of the Mother** 68.8%

Illiterate
Can read and write
Primary school
Middle school/secondary school College degree
Post-graduate degree

**The total income of the family per month**

less than 1000 US Dollar

1000 - 1999 US Dollar

2000- 3999 US Dollar

4000 - 5999 US Dollar

More than 6000 US Dollar

**Citizenship**

Citizen

Resident

Other

95.8%

**What are the current restrictive measures in the country?**

Partial lockdown

Full lockdown

No lockdown

**Days since the start of the lockdown**

less than 28 days
4 weeks to 8 weeks

8 weeks to 12 weeks

more than 12 weeks

**Employment status during COVID 19**

My current work terminated
unpaid leave
paid leave
working regular hours

working less hours

working more hours

**Parent psychiatric history**

**Have you or the other parent ever been diagnosed with a mental disorder?**

Yes
No
Prefer not to answer7.5%

10.4%

**Parent psychiatric history (disorders)**

**Please specify, you can choose more than one**

Depression

Sleep disorders
Anxiety disorder

Bipolar disorder

Psychosis Autism

ADHD

Intellectual disability (mild)

Learning disabilities

Other


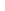


**Child information**

**Age of the child**

8-11 and 9 months

12-15 and 9 months

16-18 and 9 months

**Gender of the child60**.4% %

Male

Female

**Schooling of the child**

grade 1-2

Grade 3-6

Grade 7-10

Grade 11-12

**Did your child felt stressed at home since the time of the pandemic outbreak ?**

Yes

No

**Was there any involvement of your child with COVID 19 cases?**

Yes

No

**What was the involvement of your child with COVID cases?**

Himself /Herself was infected

One of his/her parents was infected

One of the siblings was infected Relative was infected
Friend was infected

**What was the severity of the cases that your child dealt with?**

Mild

Moderate

Severe (ICU admission)

Death

**Child's psychiatric history**

**Has your child ever been diagnosed with any mental disorder?**

yes
No
Prefer not to answer

7.3%

**Child's psychiatric history (disorders)**

**Screen for Child Anxiety Related Disorders (SCARED)PARENT Version (to be filled out by the PARENT)**

**Please specify, you can choose more than one**

Depression

Anxiety disorder

Sleep disorders

Bipolar disorder

Psychosis Autism ADHD Learning disabilities Intellectual disability (mild)

Elimination disorders

Other
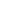


**1. When my child feels frightened, it is hard for him/her to breathe**

Not True or Hardly Ever True (0)

Somewhat True or Sometimes True (1)

Very True or Often True (2)

**2. My child gets headaches when he/she are at school.** 72.9%

Not True or Hardly Ever True (0)

Somewhat True or Sometimes True (1)

Very True or Often True (2)

**3. My child doesn’t like to be with people he/she does't know well.**

Not True or Hardly Ever True (0)

Somewhat True or Sometimes True (1)

Very True or Often True (2)

49%

**4. My child gets scared if he/she sleeps away from home.** 14.6%

Not True or Hardly Ever True (0)

Somewhat True or Sometimes True (1)

Very True or Often True (2)

**5. My child worries about other people liking him/her.**

Not True or Hardly Ever True (0)

Somewhat True or Sometimes True (1)

Very True or Often True (2)

**6. When my child gets frightened, he/she feels like passing out.**

Not True or Hardly Ever True (0)

Somewhat True or Sometimes True (1) 96.9%

Very True or Often True (2)

**7. My child is nervous.**

Not True or Hardly Ever True (0)

Somewhat True or Sometimes True (1)

Very True or Often True (2)

**8. My child follows me wherever I go.**

Not True or Hardly Ever True (0)

Somewhat True or Sometimes True (1)

Very True or Often True (2)

**9. People tell me that my child looks nervous.**

Not True or Hardly Ever True (0)

Somewhat True or Sometimes True (1)

Very True or Often True (2)

%

**10. My child feels nervous with people he/she doesn’t know well.**

Not True or Hardly Ever True (0)

Somewhat True or Sometimes True (1)

Very True or Often True (2)

**11. My child gets stomachaches at school.**

Not True or Hardly Ever True (0)

Somewhat True or Sometimes True (1)

Very True or Often True (2)

7.3%

**12. When my child gets frightened, he/she feels like he/she is going crazy.**

Not True or Hardly Ever True (0)

Somewhat True or Sometimes True (1)

Very True or Often True (2)80.2%

**13. My child worries about sleeping alone.**

Not True or Hardly Ever True (0)

Somewhat True or Sometimes True (1)

Very True or Often True (2)

**14. My child worries about being as good as other kids.** 37.5%

Not True or Hardly Ever True (0)

Somewhat True or Sometimes True (1)

Very True or Often True (2)

**15. When my child gets frightened, he/she feels like things are not real.**

Not True or Hardly Ever True (0)

Somewhat True or Sometimes True (1)

Very True or Often True (2)

89.6%

**16. My child has nightmares about something bad happening to his/her parents.**

Not True or Hardly Ever True (0)

Somewhat True or Sometimes True (1)

Very True or Often True (2)

**17. My child worries about going to school.** 5%

Not True or Hardly Ever True (0)

Somewhat True or Sometimes True (1)

Very True or Often True (2)

**18. When my child gets frightened, his/her heart beats fast.**

Not True or Hardly Ever True (0)

Somewhat True or Sometimes True (1)

Very True or Often True (2)

**19. He/she child gets shaky.%**

Not True or Hardly Ever True (0)

Somewhat True or Sometimes True (1)

Very True or Often True (2)

**20. My child has nightmares about something bad happening to him/her.**

Not True or Hardly Ever True (0)

Somewhat True or Sometimes True (1)

Very True or Often True (2)

69.8%

**21. My child worries about things working out for him**

Not True or Hardly Ever True (0)

Somewhat True or Sometimes True (1)

Very True or Often True (2)

**22. When my child gets frightened, he/she sweats a lot.**

Not True or Hardly Ever True (0)

Somewhat True or Sometimes True (1)

Very True or Often True (2)

**23. My child is a worrier.**

Not True or Hardly Ever True (0)

Somewhat True or Sometimes True (1)

Very True or Often True (2)

49%

**24. My child is really frightened for no reason at all.**

Not True or Hardly Ever True (0)

Somewhat True or Sometimes True (1)

Very True or Often True (2)

**25. My child is afraid to be alone in the house.**

Not True or Hardly Ever True (0)

Somewhat True or Sometimes True (1)

Very True or Often True (2)

**26. It is hard for my child to talk with people he/she doesn’t know well.**

Not True or Hardly Ever True (0)

Somewhat True or Sometimes True (1)

Very True or Often True (2)

**27. When my child gets frightened, he/she feels like he/she is choking.**

Not True or Hardly Ever True (0)

Somewhat True or Sometimes True (1)

Very True or Often True (2)

**28. People tell me that my child worries too much.**

Not True or Hardly Ever True (0)

Somewhat True or Sometimes True (1)

Very True or Often True (2)

79.2%

**29. My child doesn't like to be away from his/her family**

Not True or Hardly Ever True (0)

Somewhat True or Sometimes True (1)

Very True or Often True (2)

60.4%

**30. My child is afraid of having anxiety (or panic) attacks.**

Not True or Hardly Ever True (0)

Somewhat True or Sometimes True (1)

Very True or Often True (2)

**31. My child worries that something bad might happen to his/her parents**

Not True or Hardly Ever True (0)

Somewhat True or Sometimes True (1)

Very True or Often True (2)

**32. My child feels shy with people he/she doesn’t know well.**

Not True or Hardly Ever True (0)

Somewhat True or Sometimes True (1)

Very True or Often True (2)

57.3%

**33. My child worries about what is going to happen in the future.**

Not True or Hardly Ever True (0)

Somewhat True or Sometimes True (1)

Very True or Often True (2)

**34. When my child gets frightened, he/she feels like throwing up.**

Not True or Hardly Ever True (0)

Somewhat True or Sometimes True (1)

Very True or Often True (2)

**35. My child worries about how well he/she does things.**

Not True or Hardly Ever True (0)

Somewhat True or Sometimes True (1)

Very True or Often True (2)

45.8%

**36. My child is scared to go to school.**

Not True or Hardly Ever True (0)

Somewhat True or Sometimes True (1)

Very True or Often True (2)

**37. My child worries about things that have already happened.**

Not True or Hardly Ever True (0)

Somewhat True or Sometimes True (1)

Very True or Often True (2)

59.4%

**38. When my child gets frightened, he/she feels dizzy.**

Not True or Hardly Ever True (0)

Somewhat True or Sometimes True (1)

Very True or Often True (2)

90.6%

8.3%

**39. My child feels nervous when he/she is with other children or adults and he/she has to do something while they watch him/her (for example: read aloud ,speak ,play a game, play a sport)..**5%

Not True or Hardly Ever True (0)

Somewhat True or Sometimes True (1)

Very True or Often True (2)

**40. My child feels nervous when he/she is going to parties, dances, or any place where there will be people that she/he doesn’t know well.**1%

Not True or Hardly Ever True (0)

Somewhat True or Sometimes True (1)

Very True or Often True (2)

**41. My child is shy**

Not True or Hardly Ever True (0)

Somewhat True or Sometimes True (1)

Very True or Often True (2)

**Strength and Difficulties Questionnaire**

**Did any of the problems above in this section started during the Pandemic COVID-19**

Yes

No

**1. My child is considerate of other people's feelings** 5% 36.5%

Not True

Somewhat True

Certainly True

**2. My child is restless, overactive, cannot stay still for long**

Not True

Somewhat True

Certainly True

**3. My child often complains of headaches, stomach-aches or sickness**

Not True

Somewhat True

Certainly True

**4. My child shares readily with other children (treats, toys, pencils, etc.)**

Not True

Somewhat True

Certainly True

2%

**5. My child often has temper tantrums or hot tempers**

Not True

Somewhat True

Certainly True

**6. My child is rather solitary, tends to play alone**

Not True

Somewhat True

Certainly True

12.5%

60.4%

**7. My child is generally obedient, usually does what adults request**

Not True

Somewhat True

Certainly True

**8. My child has many worries, often seems worried**

Not True

Somewhat True

Certainly True

**9. My child is helpful if someone is hurt, upset or feeling ill**

Not True

Somewhat True

Certainly True

**10. My child is onstantly fidgeting or squirming**

Not True

Somewhat True

Certainly True

**11.My child as at least one good friend**

Not True

Somewhat True

Certainly True

**12. My child often fights with other children or bullies them**

Not True

Somewhat True

Certainly True

89.6%

**13. My child is often unhappy, down-hearted or tearful**

Not True

Somewhat True

Certainly True

**14. My child is generally liked by other children**

Not True

Somewhat True

Certainly True

**15.My child is easily distracted, concentration wanders**

Not True

Somewhat True

Certainly True6%

42.7%

**16. My child is nervous or clingy in new situations, easily loses confidence**

Not True

Somewhat True

Certainly True

**17. My child is kind to younger children**

Not True

Somewhat True

Certainly True

**18. My child often lies or cheats**

Not True

Somewhat True

Certainly True

78.1%

**19. My child is picked on or bullied by other children**

Not True

Somewhat True

Certainly True

**20. My child often volunteers to help others (parents, teachers, other children)**

Not True

Somewhat True

Certainly True

**21. My child thinks things out before acting**

Not True

Somewhat True

Certainly True

18.8%

**22. My child steals from home, school or elsewhere**

Not True

Somewhat True

Certainly True

96.9%

**23. My child gets on better with adults than with other children**

Not True

Somewhat True

Certainly True

**55.2%**

**24. My child has many fears, is easily scared**

Not True

Somewhat True

Certainly True

**25. My child sees tasks through to the end, he/she has a good attention span**

Not True

Somewhat True

Certainly True

29.2%

**Overall, do you think that your child has difficulties in one or more of the following areas: emotions, concentration, behavior, or being able to get on with other people?**

No
Yes minor difficulties

Yes definite difficulties

Yes severe difficulties

13.5%

42.7%

**Specifics on difficulties**

**How long have these difficulties been present?**

Less than a month 1-5 months
6-12 months
Over a year

12.7% 14.5%

**Do the difficulties upset or distress your child?7**.3%

Not at all

Only alittle

Quite a lot

A great deal

**Do the difficulties interfere with your child's everyday life in the following areas?**

Not at all

Only alittle

Quite a lot

A great deal

**Do the difficulties put a burden on you or the family as a whole?**

Not at all

Only alittle

Quite a lot

A great deal

**Have these difficulties got worse during COVID 19 Pandemic?**

Not at all

Only alittle

Quite a lot

A great deal


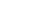

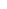

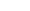

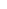

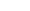

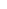


**Difficulties onset during the pandemic**

Did any of these difficulties start during the COVID 19 pandemic?

Yes
No

**Categories of difficulties starting during pandemic What kind of difficulties started during the pandemic COVID 19? you can choose more than one**

Low

sad mood

reactivity and tantrums Attention and concentration behavior

getting along with others

Other

**Coping Strategies، time on electronics and parent-child relationship**

**Since the beginning of COVID 19 have you practiced any coping strategies for dealing with stress as a family, individually or just your child?**

You can choose more than one.

Religious rituals

Exercise

Meditations

Book readings

goal setting

Planting plants and writing poetry
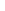

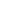

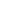

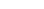


**Video gaming and screen time**


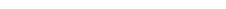


**On average, how many hours did your child spend on screens prior to COVID 19 pandemic?**

2 hours a day or less

2 - 4 hours a day

4-6 hours a day

6-8 hours a day

more than 8 hours a day

**On average, how many hours does your child spend on screens since the start of the COVID 19 pandemic?** .5%

2 hours a day or less
2 - 4 hours a day
4-6 hours a day
6-8 hours a day
more than 8 hours a day

**How would you describe your relationship with your child during COVID 19 pandemic?**

29.2%

Increased tension and frustration

More relaxed No change

Thank you for your participation please click submit, then fill another form for another child if you have more than one child by following the link again
